# Development of a Wearable Ultrasound Transducer for Sensing Muscle Activities in Assistive Robotics Applications

**DOI:** 10.3390/bios13010134

**Published:** 2023-01-13

**Authors:** Xiangming Xue, Bohua Zhang, Sunho Moon, Guo-Xuan Xu, Chih-Chung Huang, Nitin Sharma, Xiaoning Jiang

**Affiliations:** 1 Joint Department of Biomedical Engineering, North Carolina State University, Raleigh, NC 27695, USA; 2 Department of Mechanical and Aerospace Engineering, North Carolina State University, Raleigh, NC 27695, USA; 3 Department of Biomedical Engineering, National Cheng Kung University, Tainan 70101, Taiwan

**Keywords:** wearable ultrasound transducer, flexible ultrasound transducer, muscle movement, robotic prosthesis, powered exoskeleton, ultrasound imaging

## Abstract

Robotic prostheses and powered exoskeletons are novel assistive robotic devices for modern medicine. Muscle activity sensing plays an important role in controlling assistive robotics devices. Most devices measure the surface electromyography (sEMG) signal for myoelectric control. However, sEMG is an integrated signal from muscle activities. It is difficult to sense muscle movements in specific small regions, particularly at different depths. Alternatively, traditional ultrasound imaging has recently been proposed to monitor muscle activity due to its ability to directly visualize superficial and at-depth muscles. Despite their advantages, traditional ultrasound probes lack wearability. In this paper, a wearable ultrasound (US) transducer, based on lead zirconate titanate (PZT) and a polyimide substrate, was developed for a muscle activity sensing demonstration. The fabricated PZT-5A elements were arranged into a 4 × 4 array and then packaged in polydimethylsiloxane (PDMS). In vitro porcine tissue experiments were carried out by generating the muscle activities artificially, and the muscle movements were detected by the proposed wearable US transducer via muscle movement imaging. Experimental results showed that all 16 elements had very similar acoustic behaviors: the averaged central frequency, −6 dB bandwidth, and electrical impedance in water were 10.59 MHz, 37.69%, and 78.41 Ω, respectively. The in vitro study successfully demonstrated the capability of monitoring local muscle activity using the prototyped wearable transducer. The findings indicate that ultrasonic sensing may be an alternative to standardize myoelectric control for assistive robotics applications.

## 1. Introduction

More than 185,000 individuals receive amputations of upper or lower limbs in the United States annually. As a result of trauma, aging, dysvascular disease, and other conditions, more than 3.6 million individuals are expected to live with amputation by 2050 [1]. Those with mobility impairments will require assistive robotics to support a high-quality, active, and productive daily life. Assisted robotics, including robotic prostheses and exoskeletons, are devices that integrate sensing, information processing, and provide assistance to individuals with disabilities [2]. The majority of commercial assistive robotics are currently equipped with mechanical sensors that detect motion and require the users to manually switch the ambulation mode of the device [3]. When users’ physical abilities are limited, the requirement for manual control can be burdensome. Therefore, dynamic measurements of voluntary muscle activity signals are necessary for controlling active assistive devices [4]. Jayarama et al. investigated the effects of powered knee–ankle microprocessor-controlled prostheses with a camera motion capture system to record the kinematics, and the use of electromyographic sensors to measure muscle activation in transfemoral amputees [5]. A multifunctional device for sensorimotor prosthetic control was developed, which consisted of electrodes for electrotactile stimulation and muscle activity measurement using electromyography (EMG) signals [6]. Sensors for measuring voluntary muscle activity signals should be non-invasive, highly effective, and wearable to improve the functionality of assistive robotics [7].

Surface electromyography (sEMG) measures the electrical activity of muscles from the surface of the skin [8] and is one of the principal non-invasive techniques to represent human voluntary motion and movement intention [9,10]. However, sEMG has some inherent limitations, such as a low signal-to-noise ratio (SNR), an inability to reliably monitor the deeper muscles, the degradation of signal due to fatigue, insufficient ability to distinguish relative muscle firings between adjacent muscles, also known as muscle crosstalk [11], and the electromechanical delay (EMD), which is defined as the time lag between the onset of electrical activation and the beginning of force production [12]. Several limitations of sEMG can be avoided through implantable electromyography (EMG) [13,14] and targeted muscle reinnervation strategies [15,16]. However, these approaches are invasive.

Medical ultrasound (US) has been used widely for soft tissue imaging due to its non-invasive and real-time abilities [17,18,19,20]. It provides anatomical information about tissues as well as functional information, such as tissue displacement, elasticity, and tissue movement velocity [21,22,23]. Due to the fact that US waves can reach deeper regions of the human body in a specific scanning location, several limitations of sEMG in detecting muscle activity can be avoided. In addition, another advantage of US imaging for detecting muscle activity is that it is not affected by muscular fatigue due to its function of providing information on the position rather than force [24]. For instance, Akhlaghi et al. presented ultrasound-based sensing models for hand motion classification [25]. US data were collected for hand prosthetic control [24] and used as an indicator of muscle fatigue during functional electrical stimulation (FES) [26,27]. Furthermore, Zhang et al. investigated the use of US-derived neuromuscular signals and sEMG signals to predict voluntary ankle dorsiflexion and evaluated the accuracy of model-based and model-free predictions [28,29]. M.-Y. Wang et al. also used US imaging to detect finger skeletal muscle movement via vector Doppler imaging [30]. However, the aforementioned studies employed conventional US probes that lack wearability, are rigid, and may not conform to a limb. Therefore, they are not suitable for continuous monitoring and control of assistive robotics.

With the progress of materials science and fabrication techniques, wearable devices have become one of the most promising medical applications [31], particularly for US imaging. Several biomedical applications of the wearable US devices are gaining popularity, including fetal heart rate monitoring [32], blood pressure monitoring [33,34], brain imaging [35], intravascular ultrasound (IVUS) imaging [36], neural modulation [37,38], local pulse wave velocity monitoring [39], and blood flow measurement [40]. The flexibility of the US transducer is very important for wearable US devices to detect muscle activity since the skin surface is not always flat over a large area. Recently, some studies have demonstrated that flexible silicon structures based on polymer-coated trenches had a high potential for flexible transducer fabrication [41,42]. Several research groups have also demonstrated that polymer-based structures can be served as flexible carriers for micro-electromechanical systems (MEMS) sensors and piezoelectric transducers [36,38,43,44,45]. However, there is no report about using a wearable/flexible transducer to detect muscle activities for assistive robotics to date.

The purpose of this study is to develop a novel wearable US device, consisting of a 4 × 4 PZT-5A array transducer and biomedical-grade polydimethylsiloxane (PDMS) for the substrate. This array was used for real-time inspection of muscle activities in vitro and in vivo. It is a promising wearable US device that will be effective in biomedical applications for the robotic prosthesis. Major novel contributions of the paper are as follows:

- The design and fabrication procedure of a flexible US transducer incorporating PZT-5A elements into PDMS substrate was demonstrated. Consequently, the proposed US transducer exhibits high flexibility and wearability.
- Sizes of PDMS substrates and transducer arrangements can be easily customized to fit the location and shape of the target muscles. As a result of the customization of the US transducer, it is possible to measure multiple muscle groups simultaneously and individually.
- A proof-of-concept study has established that the wearable device can accurately monitor muscle movements both regionally and at different depths.

## 2. Materials and Methods

### 2.1. Transducer Design and Fabrication

**Figure 1:**
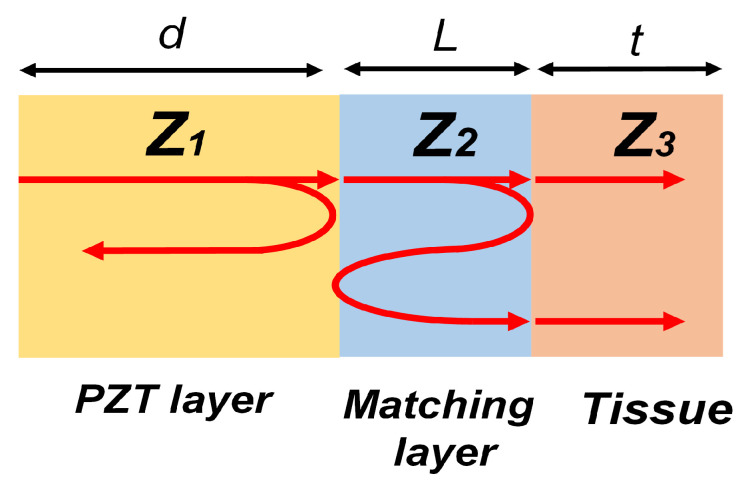
A schematic representation of wave transmission and reflection in the matching layer.

The main components of a single-element transducer are the matching layer, the active layer, and the backing layer. Figure 1 illustrates the typical structure of a piezoelectric ultrasound transducer where $z_{1}$, $z_{2}$, and $z_{3}$ indicate the acoustic impedance of piezoelectric material, matching layer, and water medium, respectively.

For the single matching layer in this design, the intensity transmission coefficient $T_{I}$ is expressed as Equation (1), where $k_{2}$ and *L* are the wave number and the thickness of the matching layer, respectively.

(1) $$T_{I}=\frac{4}{2+\left(\frac{z_{3}}{z_{1}}+\frac{z_{1}}{z_{3}}\right)\cos^{2}\left(k_{2}L\right)+\left(\frac{z_{2}^{2}}{z_{1}z_{3}}+\frac{z_{1}z_{3}}{z_{2}^{2}}\right)\sin^{2}\left(k_{2}L\right)}$$

If the thickness of the matching layer is the quarter wavelength, this transmission coefficient can be simplified as Equation (2),

(2) $$T_{I}=\frac{4z_{1}z_{3}}{{\left(z_{2}+\frac{z_{1}z_{3}}{z_{2}}\right)}^{2}}$$

If the acoustic impedance of the matching layer $z_{2}$ is equal to the geometric mean value as expressed in Equation (3), the maximum wave transmission is achieved [46].

(3) $$z_{2}=\sqrt{z_{1}z_{3}}$$

Therefore, the transducer design with a quarter-wavelength acoustic matching layer increases the transmitting power and sensitivity with wide bandwidth. In this design, the Alumina powders mixed with epoxy are used as the matching materials, in which the acoustic impedance can be adjusted by changing the weighting ratio between powders and epoxy.

The active layer is the most important part of the US transducer. The thickness of the active layer *L* is the half wavelength in the materials as expressed in Equation (4).

(4) $$L=\frac{\lambda }{2}$$

The small structure, cost-effectiveness, and linear transmission properties of PZT materials make them ideal for sensors, actuators, and transducers. Its characteristics have led to its wide use in a variety of industrial and biomedical applications [34,47]. PZT-5A is characterized by a high electromechanical coupling coefficient and a high piezoelectric coefficient [48], providing it with high pulse-echo sensitivity, which is an important characteristic when it comes to active materials.

The backing layer design aims to minimize the signal from the back surface and reduce the ring-down signal. Therefore, to reduce the wave’s capacity to travel in the opposite direction, it is always necessary to place an acoustically lossy material on the back of the piezoelectric layer to dampen the energy. If there is enough attenuation, the reflections from the back surface of the backing layer can be reduced to a minimal level, and the noise it produces can be eliminated. It is possible, in principle, to match the acoustic impedance of the backing layer with that of the piezoelectric material to reduce the amount of ring-down signal. However, the wave traveling in the opposite direction will experience complete damping, resulting in a higher bandwidth but a lower amplitude for the transducer. As a rule, the backing layer is comprised of a substance that is chosen due to its relatively high acoustic impedance and attenuation coefficients. Examples of such substances are tungsten/epoxy and silver/epoxy used in our design.

When polymers are used in flexible systems such as flexible ultrasound transducers, compatibility problems may arise. It is important that the fabrication process does not suffer from the inherent flexibility of polymers. Wearable ultrasound transducers for sensing muscle activity will require a high degree of flexibility as well as a certain level of mechanical rigidity. Within this framework, the capability to fine-tune the mechanical characteristics of such polymers is essential. The elastic modulus of PDMS is typically less than one megapascal, making it a very flexible and practical soft polymeric material [49]. The fact that the estimated modulus of PDMS is lower than those of the vast majority of polymers is evidence that directly demonstrates the material’s exceptional degree of flexibility at the macroscopic level [50]. In addition, PDMS is an excellent material to combine with rigid ultrasonic transducers since it is chemically stable and resistant to corrosion [51]. As a wearable ultrasonic transducer substrate, it is a suitable choice due to its ease of manufacture, optical transparency, biocompatibility, and low cost.

**Figure 2:**
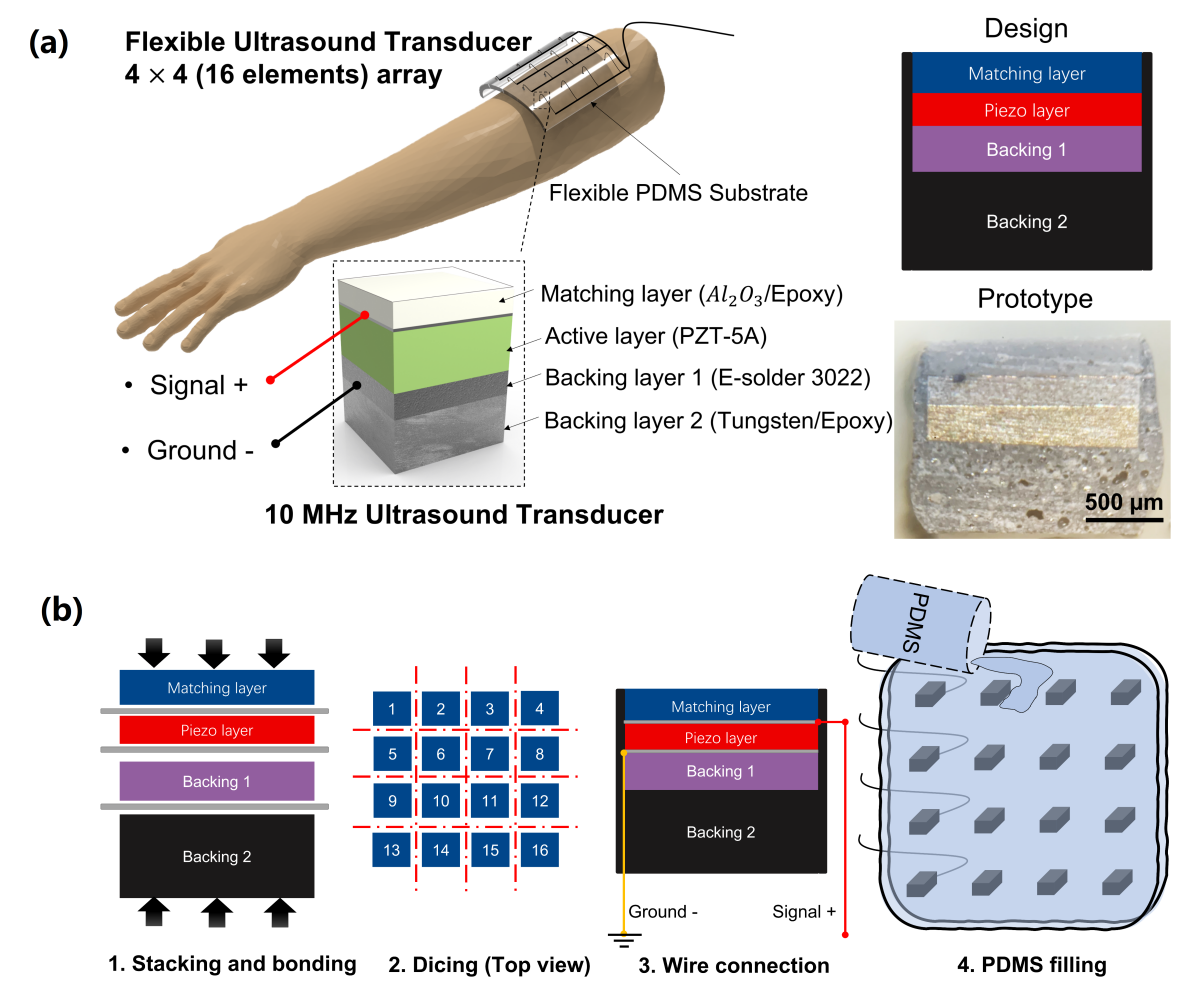
(**a**) Schematic demonstration of the wearable US transducer design. (**b**) The fabrication process for the wearable US transducer.

**Figure 3:**
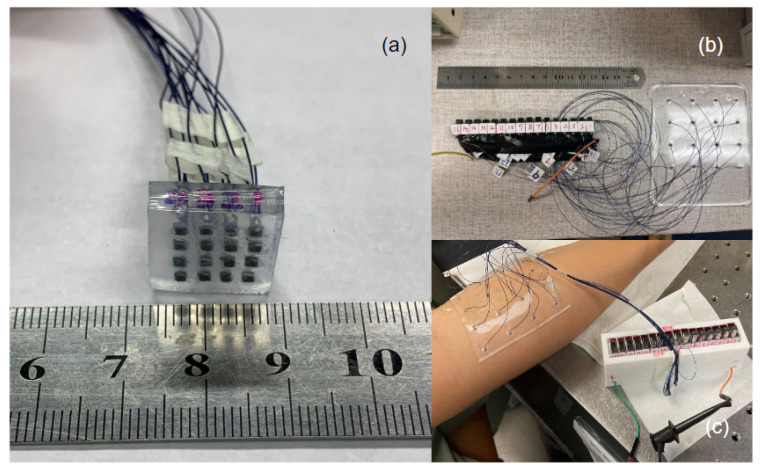
Demonstration of the wearability and customizability of the transducer: (**a**) size is about 1.5 cm, which can be used for single muscle measurements; (**b**) Size is about 10 cm, which can be used for multiple muscles measurements; (**c**) demonstration of flexibility for the wearable transducer.

A schematic demonstration of the wearable US transducer is shown in Figure 2a. The wearable US array was designed to have 16 elements in this study. Each element was individually wire connected to a coaxial cable to reduce the potential wire damage during any bending or extended motion. Due to the depth of penetration required for muscle movement detection, each element had a center frequency of 10 MHz. The 10 MHz elements consisted of one PZT-5A (Chengdu Chengyao Technology Co., Ltd. Chengdu, Sichuan, China) piezo ceramic plate (lateral size: 1.4 mm and thickness: 0.2 mm) which were diced mechanically and lapped down to obtain the desired thickness. An acoustic matching layer (thickness: 0.25 mm) made of aluminum oxide/epoxy (particle size: 50 nm) was attached to the lapped piezo layer by epoxy (EpoTek 301, Epoxy Tech. Inc., San Jose, CA, USA). The electrically conductive epoxy (E-Solder 3022, Von-Roll Inc., Cleveland, OH, USA) was attached to the back side of the piezo layer as the first backing layer (thickness: 0.28 mm). Additionally, the mixture of epoxy with tungsten particle was cast to the first backing layer as a second backing layer. The fabrication process of the wearable US transducer was mainly divided into four steps, as shown in Figure 2b. First, the matching layer, piezo layer, silver/epoxy backing layer, and tungsten/epoxy backing layer were stacked and bonded with EpoTek 301 epoxy. Second, the bonded stacks were diced into individual small elements (lateral size: 1.4 mm). Third, the coaxial cable was connected to each element. The ground connection was implemented onto the electrode placed on the backside of the piezo layer with an E-Solder 3022 epoxy. The positive cable was carefully bonded to the electrode of the front side of the piezo layer with a conductive epoxy. After the wire connection, the elements were coated with parylene-C (SCS Labcoter, PDS 2010, SCS, Indianapolis, IN, USA) to provide a protective layer. Finally, the fabricated elements were attached to the square shape 3D-printed mold (length: 100 mm, width: 80 mm, and height: 3 mm) and arranged into a 4 × 4 array with about 18 mm between each element. The PDMS was then poured into the 3D-printed mold to form the flexible transducer array substrate. Subsequently, the PDMS-filled mold was transferred to the oven and kept at a temperature of 50 °C for 6 h to be fully cured. The photograph of the proposed transducer is shown in Figure 3.

### 2.2. Transducer Characterizations

**Figure 4:**
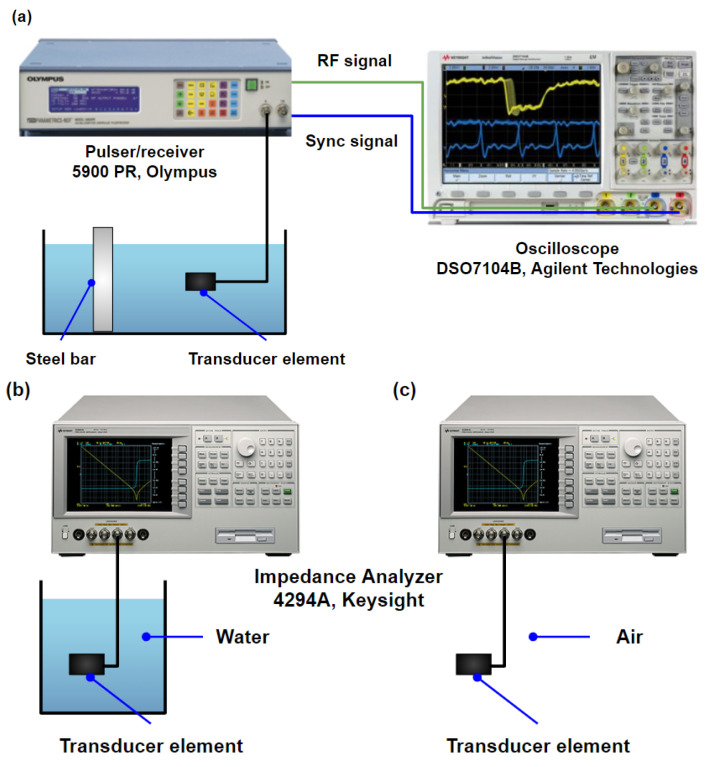
Schematics of the experimental setups for transducer characterizations: (**a**) pulse/echo test for the transducer; electrical impedance measurements for the transducer element in (**b**) water and in (**c**) air.

First, a pulse-echo test of the transducer was performed to evaluate the bandwidth and central frequency of all 16 fabricated elements, as shown in Figure 4a. A pulser/receiver (5900 PR, Olympus, WA, USA) with a pulse repetition frequency (PRF) of 200 Hz and pulse energy of 1 μJ was used to excite the elements. A bandpass filter of 3 to 20 MHz was set for receiving the echo signals. A steel bar served as the reflector. The radio-frequency (RF) signal was captured via an oscilloscope (DSO7104B, Agilent Technologies, Santa Clara, CA, USA). The bandwidth and central frequency of the fabricated elements were determined from the measured pulse-echo signal. To determine the electric impedance, capacitance, and loss, elements were connected to the impedance analyzer (4294A, Keysight Technologies, Santa Rosa, CA, USA) and measured in the air and water separately. Figure 4b illustrates the setup for electrical impedance measurements.

### 2.3. In Vitro Experimental Setup

**Figure 5:**
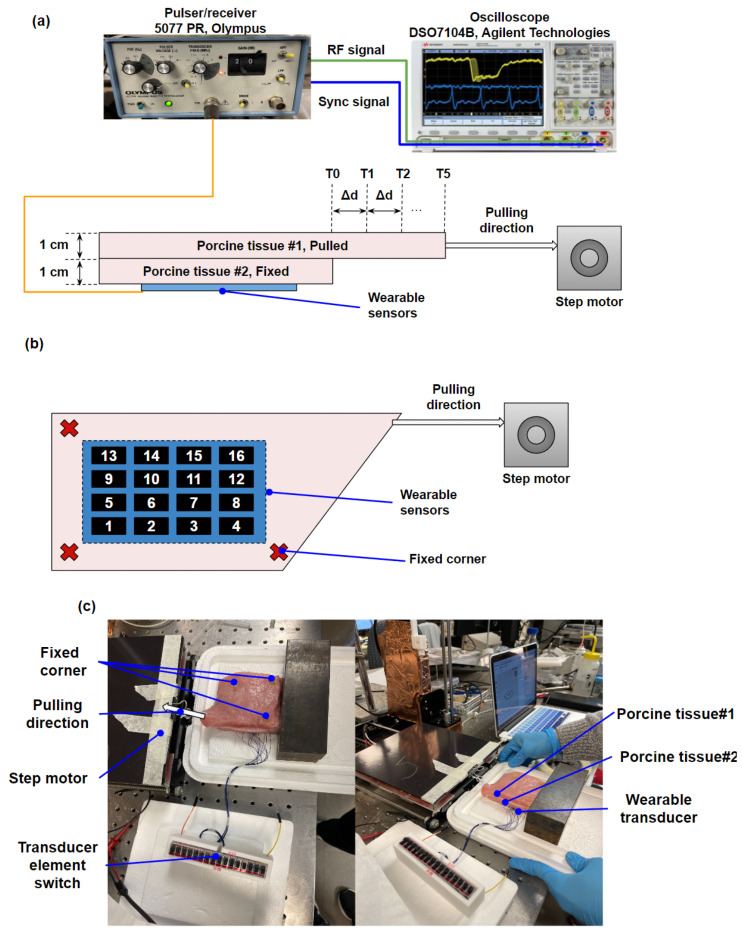
Experimental setup for in vitro test: block diagrams of the experimental system: (**a**) side view and (**b**) top view; (**c**) photographs of the experimental setup.

In vitro porcine tissue experiments were carried out to simulate muscle motions for demonstrating the performance of the wearable US device. The block diagram of the experimental system setup is shown in Figure 5. From top to bottom, porcine tissue #1 and porcine tissue #2 with a thickness of 1 cm were placed on the transducer, as shown in Figure 5a (side view). Both transducer and porcine tissue#2 were totally fixed with pins, while porcine tissue#1 was fixed at three corners. A step motor was used to apply a pulling force to the non-fixed corner of porcine tissue#1, as shown in Figure 5b (top view). The size of the porcine tissue is approximately 14 cm by 6 cm by 1 cm (L by W by H). Due to the fact that the total pulled distance of porcine tissue #1 was 5 mm, it was possible to ignore the thickness change of the porcine tissue. The experimental setup was designed to mimic the environment of human muscle. Moving porcine tissue (porcine tissue #1) could be considered a muscle fiber in the human body. Porcine tissue #2 refers to the skin of the human body. A pulser/receiver (5077 PR, Olympus, WA, USA) with a PRF of 200 Hz, a pulse energy of 2 μJ, a gain of 20 dB, and a high pass filter of 1 MHz, was used to excite the transducer while porcine tissue#1 was pulled. One corner of porcine tissue#1 was pulled by the step motor with five equal steps over a distance of 5 mm during the experiments. An oscilloscope (DSO7104B, Agilent Technologies, Santa Clara, CA, USA) was used to capture the RF signal from all 16 elements as porcine tissue#1 was pulling from T0 to T5 (T0 represents the RF signal before pulling, the distance between time intervals is 1 mm). Figure 5c shows the photos for the experimental setup. The sound velocity in a muscle tissue is typically about 1550 m/s [52]. Since the thicknesses of both porcine tissue #1 and porcine tissue #2 are 1 cm, the time intervals between porcine tissue #1 and #2 were determined from the backscattering signals of muscles to separate different muscles.

### 2.4. Preliminary In Vivo Experimental Setup

**Figure 6:**
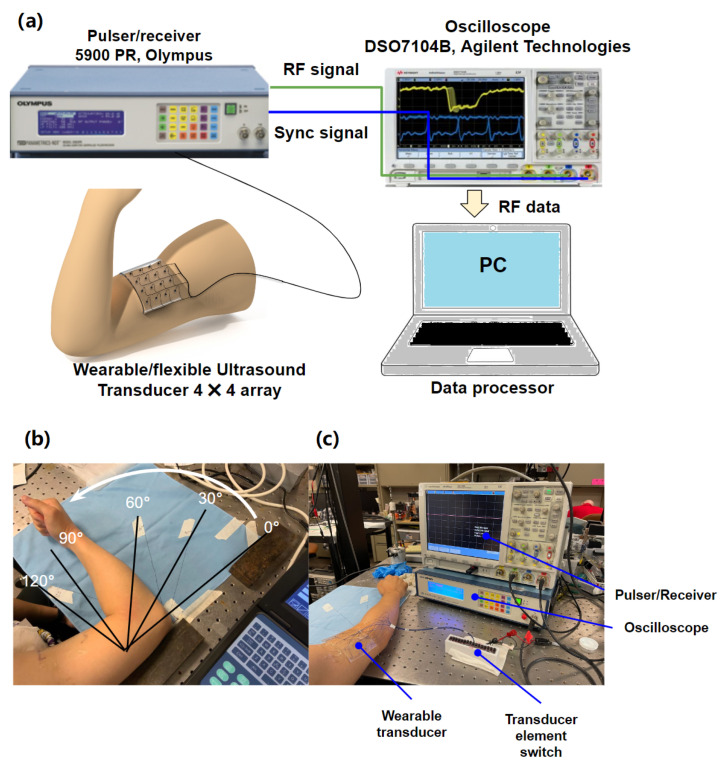
The experimental setup used for the in vivo test: (**a**) block diagrams of the experimental system; (**b**,**c**) photographs of the experimental setup.

A preliminary in vivo experiment was conducted to demonstrate the performance of using the proposed wearable transducer for detecting muscle activity in humans. Figure 6a illustrates the block diagram of the experimental system setup. As shown in Figure 6b, wearable transducers were attached to the subject’s biceps brachii muscle to detect muscle activity as the subject flexed his elbow from $0^{\circ }$ to $120^{\circ }$. Three trials were conducted in total. Each trial began with the use of a commercial B-mode ultrasound probe to ensure that the wearable transducer was tracking the muscle in the appropriate region and depth prior to the collection of US signals. A pulser/receiver (5900 PR, Olympus, WA, USA) with a pulse energy of 1 μJ, a gain of 40 dB, a high pass filter of 3 MHz, and a low pass filter of 20 MHz, was used to excite the transducer while the forearm was moved. An oscilloscope (DSO7104B, Agilent Technologies, Santa Clara, CA, USA) was used to capture the RF signal from all 16 elements as the subject flexed his elbow from $0^{\circ }$ to $120^{\circ }$ with $30^{\circ }$ intervals. The photographs for the experimental setup are shown in Figure 6c.

### 2.5. RF Data Processing Procedure

The RF data of 16 elements were acquired from the oscilloscope with a sampling rate of 4 GHz for post-processing to detect the porcine tissue displacements for the in vitro test and human muscle motions for the preliminary in vivo test of each element. All the signal and image processing were performed using MATLAB (R2020b, The MathWorks, Natick, Massachusetts, USA). First, the CSV files recorded from the oscilloscope were converted to MATLAB files. After the files were loaded, the range (length) of muscle backscattering signals was determined according to the B-mode image from a commercial US system. The backscattering signal at the initial location was referred to as the reference signal (T0), and the tissue displacement was determined at each pulling step via the normalized cross-correlation (NCC), which calculated the difference in phase between the reference signal and the comparison signal [53]:

(5) $$\mathrm{NCC}\left(t\right)=\frac{\sum_{n=1}^{W}f\left(n\right)g\left(n+t\right)}{\sqrt{\sum_{n=1}^{W}f^{2}\left(n\right)\cdot \sum_{n=1}^{W}g^{2}\left(n+t\right)}},\left(t_{1}\leq t\leq t_{2}\right)$$

where the f(n) and g(n) are the reference signal and comparison signal, respectively, *n* is the index of samples (from 1 to *W*), *W* is the window length of comparing samples, *t* is the time shift between the reference signal and the comparison signal, and $\left[t_{1},t_{2}\right]$ is the time range of interest in the reference signal. After the phase shifts of each element were determined at different pulling steps, the relative tissue displacements were then obtained since the angle between the US beam and muscle orientation is unknown. Subsequently, the relative muscle displacements were color encoded on a 4×4 matrix to show the muscle movement imaging. A linear interpolation method was applied to the matrix for image smoothing.

## 3. Results

### 3.1. Transducer Characterizations

**Figure 7:**
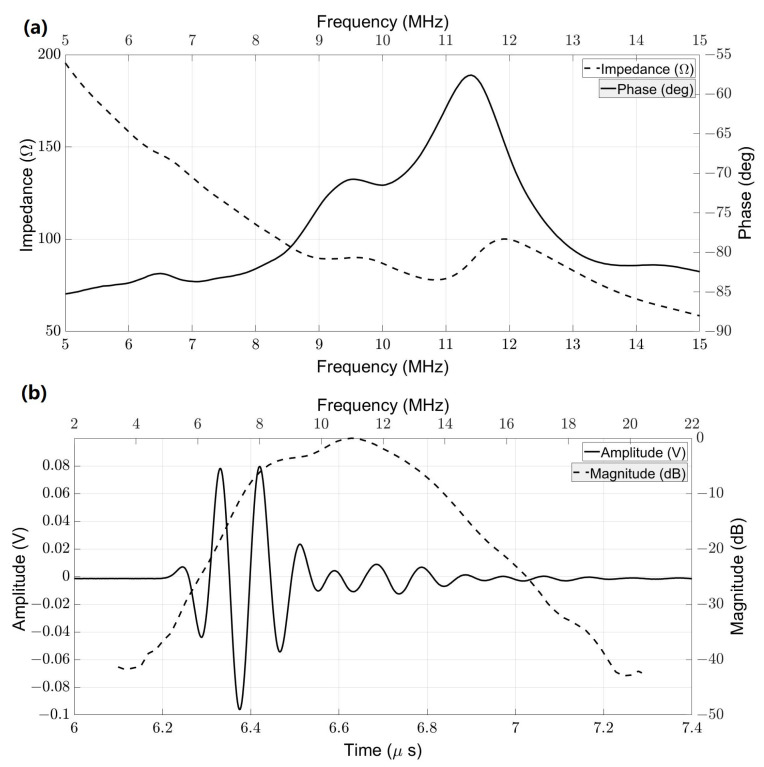
Transducer characterizations element #10: (**a**) measured electrical impedance and (**b**) pulse-echo response of element #10.

Figure 7 shows the typically measured impedance of the transducer in the water and the pulse-echo results for one of the elements. Both measured results came from element #10 as a sample. Figure 7a depicts that the central frequency was 10.61 MHz with an impedance of 75.27 Ω in the water. Capacitance and dielectric loss were 176.98 pF and 9.37 mU, respectively. Figure 7b shows the pulse-echo result for element #10. With 1 μJ pulse energy, the peak-to-peak amplitude was 175.8 mV, and the -6dB bandwidth was 48.82%. Furthermore, the characterizations, including central frequency, fractional bandwidth, loop sensitivity, capacitance, loss, and impedance of all 16 elements and their average and standard deviation were listed in Table 1. Based on the average and standard deviation values for all acoustic characteristics, we can conclude that all elements are manufactured in a consistent and efficient manner.

### 3.2. In Vitro Results

**Figure 8:**
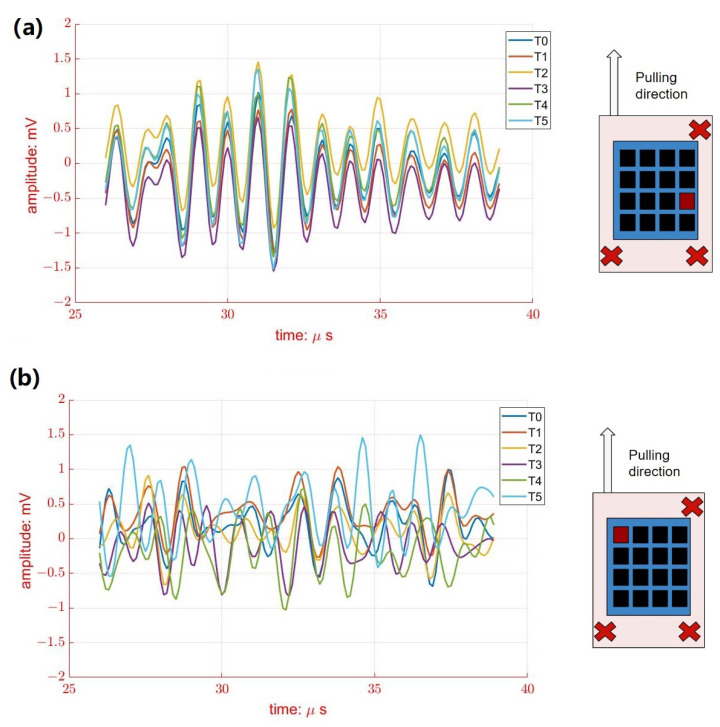
The variations of received RF signals from porcine tissue#1 at different pulling distances from T0 to T5 from (**a**) element #2 and (**b**) element #16. The location of selected elements is marked in red.

Figure 8a shows the typical ultrasonic backscattering signals of porcine tissue at element #2 from different pulling distances (from T0 to T5). Since the porcine tissue was fixed at three corners surrounding element#2, muscle displacement was limited during pulling. Therefore, it is obvious that the phases of ultrasonics backscattering signals at element #2 seem similar during pulling, as shown in Figure 8a. On the contrary, larger variations of ultrasonic backscattering signals were observed clearly at element #16 from different pulling distances (T0 to T5), as shown in Figure 8b, where the location of element #16 was close to the pulling site that exhibited a larger muscle displacement. In other words, the muscle displacements were detected regionally by all 16 elements of the wearable US transducer.

**Figure 9:**
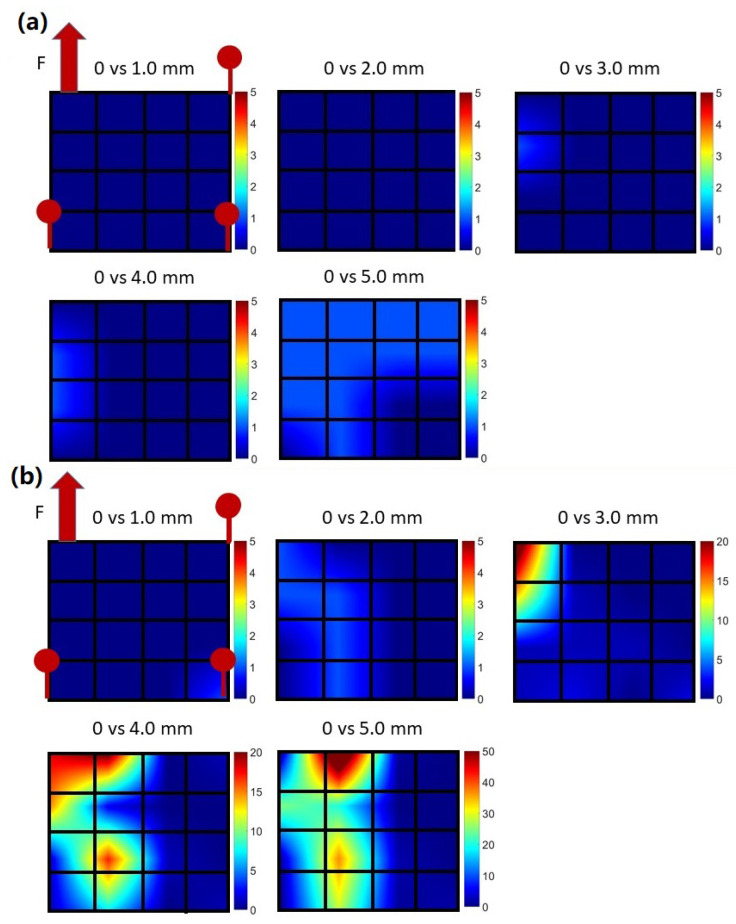
Muscle movement imaging for (**a**) porcine tissue#2 and (**b**) porcine tissue#1. The color bar represents the relative displacement of the muscle. The displacement was calculated using T0 as the reference.

Figure 9a shows the typical muscle movement imaging from porcine tissue#2 at different pulling distances. RF data from 16 elements were first acquired from the oscilloscope. According to the NCC method described in Section 2.5, which calculates the phase difference between the reference signal and the comparison signal, the relative muscle displacements were calculated by calculating the phase change of backscattering signals during pulling. The 4 × 4 matrix represents the locations of 16 elements. Since porcine tissue #2 was fixed without pulling, the relative muscle displacements seem to remain constant for all elements during the experiment. On the contrary, according to Figure 9b the muscle movement imaging from porcine tissue #1 exhibited a larger variation of relative displacement during pulling. Muscle displacements were increased with pulling, especially around the pulling site, which is in line with the experimental setup that only porcine tissue#1 was pulled during the experiment. This experimental result is evidence that the proposed wearable transducer can not only detect regional muscle displacements but also at different depths.

### 3.3. Preliminary In Vivo Results

**Figure 10:**
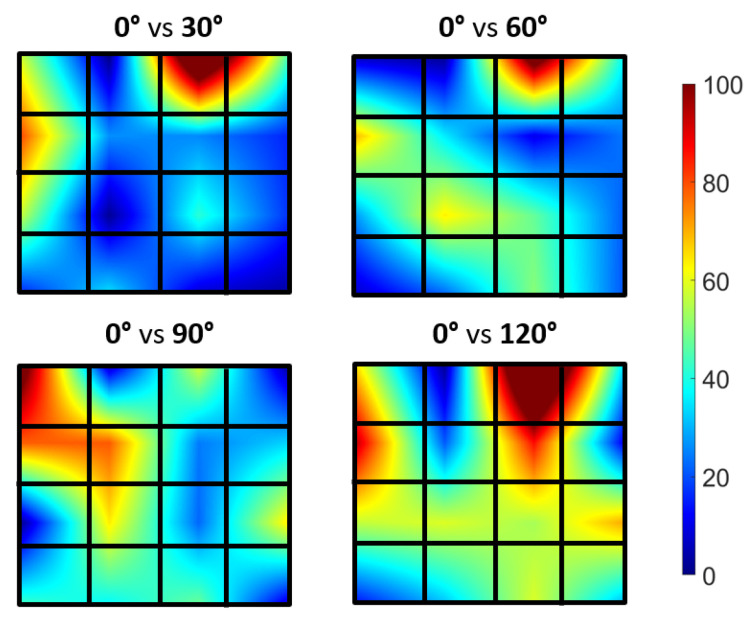
Muscle average movement imaging during in vivo test. The color bar represents the relative displacement of the muscle. The displacement was calculated using the forearm at $0^{\circ }$ as the reference.

Figure 10 illustrates the typical muscle movement images of three trials of the biceps brachii at various elbow flexion angles (average from three trials). In a similar manner to Section 3.2, the relative muscle displacements were determined by calculating the phase change of backscattering signals during forearm movement using the NCC method. The muscle displacements were obtained from the proposed wearable transducer during body movement. Additionally, there was a larger displacement in the region of the target muscle associated with the bending movement, which is represented in the color map as red. It is possible to determine the role of a specific muscle using this method.

## 4. Discussion and Future Works

In this study, we described the development of a wearable US device in which the PZT-5A elements are embedded into deformable PDMS substrates. PZT is widely used in US transducers [54,55]. The rigidity of PZT led us to select PDMS as a flexible substrate for providing mechanical interlinkage between elements. In contrast to traditional US transducers, which are based on rigid substrates, the PDMS substrate is selected for several reasons. To begin with, PDMS has a stretchability of >170% in tensile strain. In addition to its flexibility, as PDMS is biocompatible, it can be safely applied in biomedical applications in close contact with the skin [36]. Moreover, PDMS material is thermally secured at certain high temperatures over 200 °C [56], which means it is safe to use it for ultrasound transducers. PDMS has suitable electrical and acoustic properties for the ultrasound transducer. It could be essential to determine the geometric design of the flexible ultrasound transducer such as the spacing between adjacent elements, the aperture size of each element, the number of elements, and so forth [57]. Otherwise, each element could have a constructive effect on the reduced performance of the flexible array transducer when it is deformed. There may be a concern regarding the dissipation of the acoustic stack with PZT from PDMS. However, the proposed flexible ultrasound transducer array is fabricated to detect muscle movement signals. By having the large spacing between each element corresponding target muscles, it may be possible to prevent detachment issues due to the relatively lower strain applied to the transducer while it is being deformed. Furthermore, the wearable transducer is relatively thin, with a thickness of approximately 3 mm. The wires of each element are individually connected with a coaxial cable to reduce the possibility of wire damage during bending or extended motion. As a result, the device is highly stretchable and reliable, which is in good agreement with several previous studies that PDMS is suitable for many biomedical applications [38,41,42,58,59].

The conventional handheld ultrasonic transducer with a rigid housing has limits in its ability to detect muscle activity as the target muscle (upper or lower limbs) is moving dramatically. When an ultrasonic transducer is pressed against a body surface, it may inhibit the activity of underlying muscles. Additionally, the accuracy of the measurement of muscle activity may be influenced significantly as the surface of a conventional transducer does not attach to the skin very well [60]. In contrast, the flexible and wearable transducer can attach to the body area of interest without restricting the movement of the underlying tissue and preventing transducer shifting. Furthermore, the sizes of PDMS substrates, the thickness of the piezo layer, and transducer arrangements can be easily tailored to fit the location, depth, and shape of the target muscle.

The wearable transducer developed in the present study is capable of accurately monitoring muscle activity. Since the muscle activities can be detected regionally by the wearable transducer, ideas for how to use these US signals to control the robotic prosthesis will be the next works. In addition, customizable devices make it possible to measure multiple muscle groups simultaneously and individually, which is an important step in developing control schemes for high-degree-of-freedom assistive robotics. This study serves as a proof-of-concept study, which confirms that the wearable device is capable of monitoring muscle movement. Quantitative comparisons between non-wearable US transducers and the proposed wearable US system are planned in the future. Moreover, over thirty tests have been conducted for in vitro and in vivo experiments using the proposed flexible US transducer array without interruption in performance due to heat generation or dissipation. Our future work will include the detailed reliability testing of wearable transducers.

Due to the fact that the US gel would make the wearable transducer slippery without the assistance of an adhesive on the skin, another alternative should be considered for the US coupling medium. Due to the issues of irritating the skin and hygiene risks, gel-type ultrasound coupling mediums would hinder the long-term application of monitors. Dry couplants are suitable for wearable ultrasound transducers and skin interfaces [61]. Dry couplant materials have a similar acoustic impedance to water, low acoustic attenuation, and are flexible yet durable. A wearable ultrasound transducer with dry couplant does not require any coupling medium between the transducer and the skin, making it more practical for continuous monitoring of muscle activity. Another area of future research will be the development of wearable transducers with dry couplants that provide minimal discomfort on human skin for long-term monitoring.

## 5. Conclusions

This study demonstrated the fabrication procedure of incorporating PZT-5A elements into PDMS substrates, resulting in an US transducer that exhibits high flexibility and wearability. The 4 × 4 array consists of 16 elements and the size of each element was 1.4 mm × 1.4 mm with a thickness of about 1.2 mm. The central frequency, −6 dB bandwidth, and electrical impedance in water were 10.59 MHz, 37.69%, and 78.41 Ω in average for the elements. In vitro experiments and preliminary in vivo experiments demonstrated the ability of the proposed transducer for monitoring muscle activity. The muscle displacement was visualized by muscle movement imaging. The proposed device is flexible, wearable, customizable, and easily extendable to motion classification and assistive robotics in the future.

## Figures and Tables

**Table 1:** Acoustic characterizations of 16 elements.

Property	Pulse-Echo Response Test			Electrical Impedance Test			
	Central Frequency (MHz)	Fractional Bandwidth (−6 dB) %	Loop Sensitivity (dB)	Capacitance (@ 1 kHz, pF)	Loss (@ 1 kHz, mU)	Impedance in Air (Ω)	Impedance in Water (Ω)
Element #1	10.04	37.05	−39.28	199.00	9.80	79.06	78.30
Element #2	10.71	30.44	−49.82	186.50	9.90	80.53	81.35
Element #3	10.69	21.89	−39.05	199.80	9.77	76.09	72.50
Element #4	9.97	59.18	−50.27	189.70	9.37	79.64	80.83
Element #5	10.61	32.61	−38.87	194.68	10.55	79.17	79.04
Element #6	10.82	29.57	−37.42	196.27	9.70	75.07	76.23
Element #7	10.37	45.71	−45.96	193.89	10.90	81.04	86.28
Element #8	10.85	32.26	−40.74	191.54	9.70	76.50	75.46
Element #9	10.85	36.31	−35.09	199.46	10.00	76.19	73.00
Element #10	10.61	48.82	−38.02	176.98	9.37	77.94	75.27
Element #11	10.18	40.47	−37.27	194.72	10.64	74.85	75.70
Element #12	10.97	30.81	−40.47	199.50	10.20	76.92	77.75
Element #13	10.83	27.52	−35.83	191.45	10.10	79.60	80.00
Element #14	10.81	32.75	−39.42	184.60	9.90	85.88	80.43
Element #15	10.93	33.12	−37.18	184.80	10.30	85.69	79.65
Element #16	10.16	64.57	−39.42	180.10	9.10	83.36	82.81
Average	10.59	37.69	−40.26	191.44	9.96	79.22	78.41
Standard Deviation	0.33	11.55	4.53	7.13	0.48	3.45	3.65

## Data Availability

The data that support the findings of this study are available from the corresponding author, X.J., upon reasonable request.
